# Development and Validation of an Interpretable Model for Predicting Postoperative Hyperlactatemia in Young Children Following Congenital Heart Surgery

**DOI:** 10.3390/jcm15051846

**Published:** 2026-02-28

**Authors:** Yuchan Chen, Wenxin Ge, Lixin Hu, Jiaqi Chen, Yajun Chen

**Affiliations:** 1 Department of Maternal and Child Health, School of Public Health, Sun Yat-sen University, No. 74 Zhongshan 2nd Road, Yuexiu District, Guangzhou 510080, China; chenych225@mail2.sysu.edu.cn (Y.C.); gewx3@mail2.sysu.edu.cn (W.G.); hulx6@mail2.sysu.edu.cn (L.H.); chenjq239@mail2.sysu.edu.cn (J.C.); 2 Cardiac Intensive Care Unit, Guangzhou Women and Children’s Medical Center, Guangzhou Medical University, Guangdong Provincial Clinical Research Center for Child Health, Guangzhou 510623, China

**Keywords:** hyperlactatemia, congenital heart disease, pediatric cardiac surgery, machine learning, explainable machine learning, risk prediction

## Abstract

**Objectives**: Postoperative hyperlactatemia (POHL) is a common complication after pediatric cardiac surgery, yet its perioperative risk factors remain unclear. This study developed and internally validated an interpretable machine learning (ML) model to identify young children at risk for POHL. **Methods**: We retrospectively analyzed 3224 children aged 0 to 36 months from 2018 to 2023. Four ML models, including logistic regression (LR), random forest (RF), support vector machine (SVM), and eXtreme Gradient Boosting (XGBoost), were trained and validated. Model performance was assessed using discrimination, calibration, and classification metrics, and decision curve analysis evaluated clinical utility. SHapley Additive exPlanation (SHAP) provided both global and local interpretability. **Results**: Of the 3224 children, 731 (22.7%) developed POHL, with a median age of 5 months. The RF model performed best (AUC, 0.821; 95% CI, 0.787–0.854; sensitivity, 69.7%; specificity, 84.1%; Brier score, 0.146). SHAP analysis identified 8 key predictors of POHL. Established factors included cardiopulmonary bypass duration, lowest bypass temperature, epinephrine dose, and RACHS-1 category. Novel contributors comprised low body weight, reduced left ventricular end-diastolic diameter, plasma transfusion, and continued mechanical ventilation within the first 24 postoperative hours. **Conclusions**: We developed and internally validated an interpretable RF model that integrates established and novel predictors to estimate POHL risk in young children after cardiac surgery. Pending external validation, it may support earlier risk recognition and more personalized perioperative management in this high-risk pediatric population.

## 1. Introduction

Congenital heart disease (CHD) is the most prevalent congenital anomaly globally and often necessitates corrective surgery under cardiopulmonary bypass (CPB) [1,2,3,4]. Despite considerable advancements in surgical techniques and perioperative care, postoperative hyperlactatemia (POHL) remains a prevalent and clinically significant complication, affecting approximately 10% to 20% of pediatric patients after cardiac surgery [4,5,6,7,8,9,10]. Elevated lactate levels may arise from both hypoxic and non-hypoxic mechanisms and are consistently associated with multi-organ dysfunction, prolonged ventilation, and increased mortality rates [9,10,11,12]. POHL is particularly pressing in regions with large pediatric populations and high surgical demands. China, which has the world’s second-largest pediatric population, faces a substantial clinical burden from POHL [13,14], underscoring the need for reliable risk-prediction tools.

While perioperative risk factors for POHL are well documented in adults, their relevance to children is limited by developmental differences, such as immature organ function and a greater prevalence of cyanotic CHD [8,9,11,12,15,16,17]. Existing pediatric studies often group children across a wide age range (0–18 years), which obscures the unique vulnerabilities of children aged 0 to 36 months, where physiological immaturity and surgical stress intersect [9,10,11,18,19]. Moreover, many previous studies have been limited by small sample sizes and have relied on traditional regression models that assume linear relationships and fail to capture complex interactions among perioperative variables [9,10,11,19]. As a result, risk factors specific to the youngest and highest-risk subgroup of children aged 0 to 36 months remain poorly defined, and effective prediction models are still lacking [9,10,11,20,21,22].

Machine learning (ML) approaches offer significant advantages for analyzing high-dimensional data and identifying nonlinear patterns that traditional regression analysis may overlook [20,21,22]. Nevertheless, the limited interpretability of many ML algorithms has constrained their adoption in clinical settings [23]. Recent advancements, such as SHapley Additive exPlanations (SHAP), facilitate transparent interpretation of ML models by quantifying both global and patient-specific feature contributions [24,25]. This study developed and internally validated an interpretable ML model to predict POHL in children aged 0 to 36 months undergoing congenital heart surgery, using data from a large single-center cohort. The aim was to improve discrimination without sacrificing explainability by incorporating SHAP outputs, thereby supporting earlier risk stratification and informing more personalized perioperative management in this high-risk pediatric population.

## 2. Methods

### 2.1. Study Population and Data Collection

This retrospective study was conducted at Guangzhou Women and Children’s Medical Center (GWCMC), a tertiary referral hospital and regional medical center affiliated with Guangzhou Medical University in southern central China. Ethical approval was obtained from the GWCMC Ethics Committee (No. [2024]397A01; 24 October 2024), and the study was performed in accordance with the principles outlined in the Declaration of Helsinki. As the research was retrospective in design, informed consent was not required. The study design and reporting strictly adhered to the 2024 TRIPOD guidelines [26].

**Figure 1:**
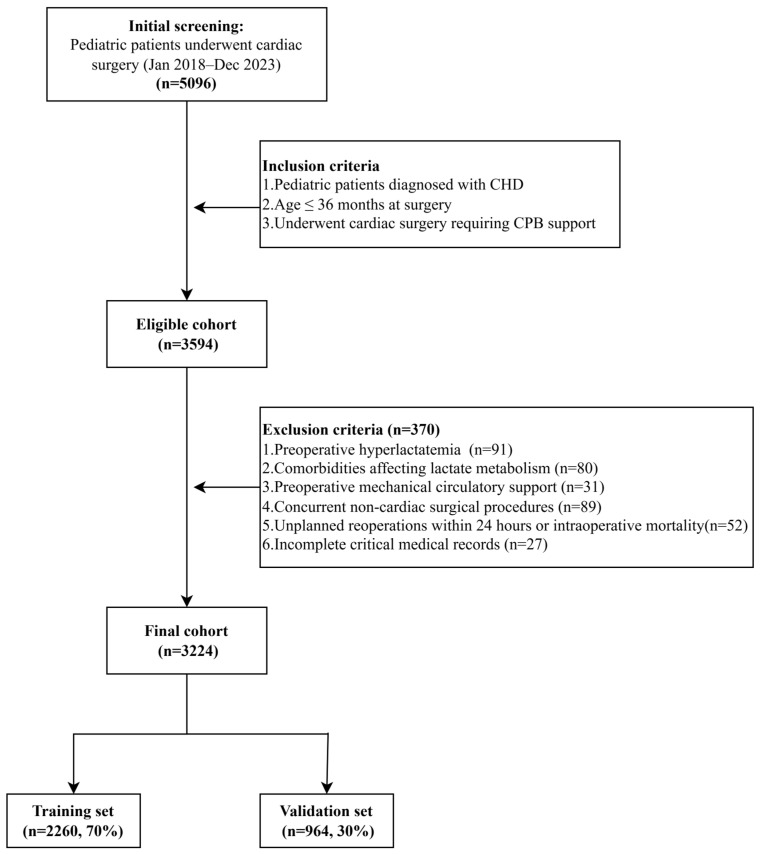
Study enrollment flowchart of pediatric patients with CHD after cardiac surgery. Abbreviations: CHD, Congenital heart disease; CPB, Cardiopulmonary bypass.

The study enrolled pediatric patients aged 36 months or younger, of both genders, diagnosed with CHD who underwent cardiac surgery with CPB support between January 2018 and December 2023. CHD diagnoses were confirmed by transthoracic echocardiography and classified according to ICD-10-CN codes (Q20–Q28). Data were extracted from the electronic medical record system using a standardized congenital heart surgery case report form (CHS-CRF), which included demographic characteristics, echocardiography findings, laboratory parameters, surgical classifications, CPB records, and postoperative treatment records. Detailed definitions and classifications of all variables are available in Supplementary Table S1. As shown in Figure 1, the initial screening identified 5096 pediatric patients who underwent cardiac surgery at GWCMC between January 2018 and December 2023. A total of 1502 patients were excluded for not meeting the inclusion criteria (CHD diagnosis, age ≤ 36 months at surgery, and CPB support), leaving 3594 eligible patients. We then excluded 370 patients for prespecified reasons: preoperative hyperlactatemia (*n* = 91), comorbidities affecting lactate metabolism (*n* = 80), preoperative mechanical circulatory support (*n* = 31), concomitant non-cardiac surgery (*n* = 89), unplanned reoperation within 24 h or intraoperative death (*n* = 52), and incomplete critical medical records (*n* = 27). The final analytical cohort included 3224 children with CHD and was randomly divided into a training set (*n* = 2260; 70%) and a validation set (*n* = 964; 30%), with randomization performed at the patient level using unique identifiers. This sample size exceeded the minimum requirement of 1260, as estimated using Riley’s four-step method (R package pmsampsize), ensuring adequate statistical power and model stability [27]. 

### 2.2. Definitions of POHL

POHL was defined as a peak arterial lactate concentration exceeding 3 mmol/L within the first 24 h following ICU admission after surgery [12,28]. Arterial lactate levels were routinely monitored according to institutional protocol: upon ICU admission (H0) and at 1, 2, 6, 12, and 24 h thereafter (H1, H2, H6, H12, H24). Additional measurements were performed at the discretion of treating physicians if clinical deterioration occurred. The highest recorded value within these 24 h was designated as the peak lactate concentration for each patient. Patients were classified into two groups: those who developed POHL and those who did not (non-POHL).

### 2.3. Data Preprocessing, Feature Selection, and Class Imbalance Handling

Outliers in continuous variables were identified using box plots. These were defined as values greater than the upper quartile plus 1.5 times the interquartile range (IQR) or less than the lower quartile minus 1.5 times the IQR. To mitigate the impact of extreme values while maintaining the overall distribution, outliers were winsorized to the nearest boundary value (Supplementary Figure S1) [29]. The extent of missing data for all variables is illustrated in Supplementary Figure S2. Variables with ≤20% missingness were retained. Categorical variables with missing values were imputed using the mode, while continuous variables with missing values were imputed using the median [30]. Categorical predictors were predefined before model construction and encoded as binary (0/1).

Feature selection utilized the Least Absolute Shrinkage and Selection Operator (LASSO) regression with L1 regularization [31]. The optimal penalty parameter (λ) was identified via 10-fold cross-validation (CV) using the one-standard-error (λ.1se) criterion to obtain a parsimonious and robust set of predictors. All feature selection procedures were conducted exclusively in the training set to prevent data leakage. The resulting feature subset was then used for model development.

To address the imbalance between the POHL and non-POHL groups in the training set, we applied a two-step hybrid resampling strategy. Initially, we oversampled the minority class (POHL) using the Synthetic Minority Over-sampling Technique (SMOTE, k = 5) to generate additional synthetic samples. Subsequently, we randomly undersampled the majority class (non-POHL) to match the minority class size, thereby creating a balanced dataset for model training. The validation set remained untouched to ensure an unbiased evaluation of model performance. A more detailed description of the resampling procedure is available in the Supplementary Materials (Supplementary Figures S3 and S4).

### 2.4. Model Development, Validation, and Interpretation

Four ML models, including logistic regression (LR), random forest (RF), support vector machine (SVM), and eXtreme Gradient Boosting (XGBoost), were employed to predict the risk of POHL in pediatric patients after cardiac surgery. We optimized model hyperparameters using grid search combined with 10 rounds of 10-fold CV on the selected feature subset [32,33]. Subsequently, the models were retrained on the whole training set using the optimal features and the tuned hyperparameters, as detailed in Supplementary Table S2.

Model performance was evaluated in the independent validation set across three main domains: discrimination, measured by the Area Under the Curve (AUC) and the Area Under the Precision-Recall Curve (AUPRC); calibration, assessed via the Brier score; and classification metrics, including accuracy, sensitivity, specificity, positive predictive value and negative predictive value, and the F1 score. For threshold-dependent measures, the probability cut-off was determined by Youden’s J statistic. The optimal model was selected based on both discrimination and calibration, with classification metrics serving as secondary confirmation. Decision curve analysis (DCA) was also conducted to assess clinical utility across a range of probability thresholds.

Model interpretability was evaluated using SHAP applied to the final selected model. Global feature importance was quantified by the mean absolute SHAP value across the cohort, while local interpretability was examined using patient-level SHAP values [23,25].

### 2.5. Statistical Analysis

Baseline characteristics were presented as means and standard deviations for normally distributed continuous variables, and as medians with IQRs for skewed variables. Categorical variables were summarized using counts and percentages. Group comparisons utilized Student’s *t*-test or Mann–Whitney U test for continuous variables, and the chi-square test for categorical variables, as appropriate. Model discrimination was evaluated using ROC and PR curves, with a greater emphasis on PR curves due to class imbalance. Calibration was assessed using the Brier score, while clinical utility was examined via DCA across clinically relevant probability thresholds. All statistical analyses were performed in R (version 4.5.1), utilizing key packages such as glmnet, caret, smotefamily, the mlr3 framework (mlr3, mlr3learners, mlr3tuning), pROC, and shapviz. A two-tailed *p*-value < 0.05 indicated significant levels. During the preparation of this work, we used ChatGPT (version 5.1) to enhance the English language and improve readability. After utilizing this tool, we thoroughly reviewed and edited the content as necessary and accept full responsibility for the final publication.

## 3. Results

### 3.1. Baseline Characteristics and Outcome Distribution

The analysis included 3224 pediatric patients who underwent cardiac surgery. Baseline characteristics were comparable between the training and validation sets, with no statistically significant differences detected (all *p* > 0.05; Table 1).

The median surgical age was 5 months (IQR, 2–11 months), and 56.4% of the cohort were boys. Most patients were term births (98.4%) and underwent elective procedures (88.9%). A significant portion received non-fast-track cardiac anesthesia management (62.8%) and were classified as ASA physical status III–VI (59.8%). In terms of surgical complexity, 66.8% of the procedures were classified as RACHS-1 categories I–II, with only 3.6% having a history of prior cardiac surgery.

The POHL occurred in 731 cases (22.7%). The incidence rates were similar between the training (*n* = 510, 22.6%) and validation (*n* = 221, 22.9%; *p* = 0.80) sets.

### 3.2. Features Selected in Models

**Figure 2:**
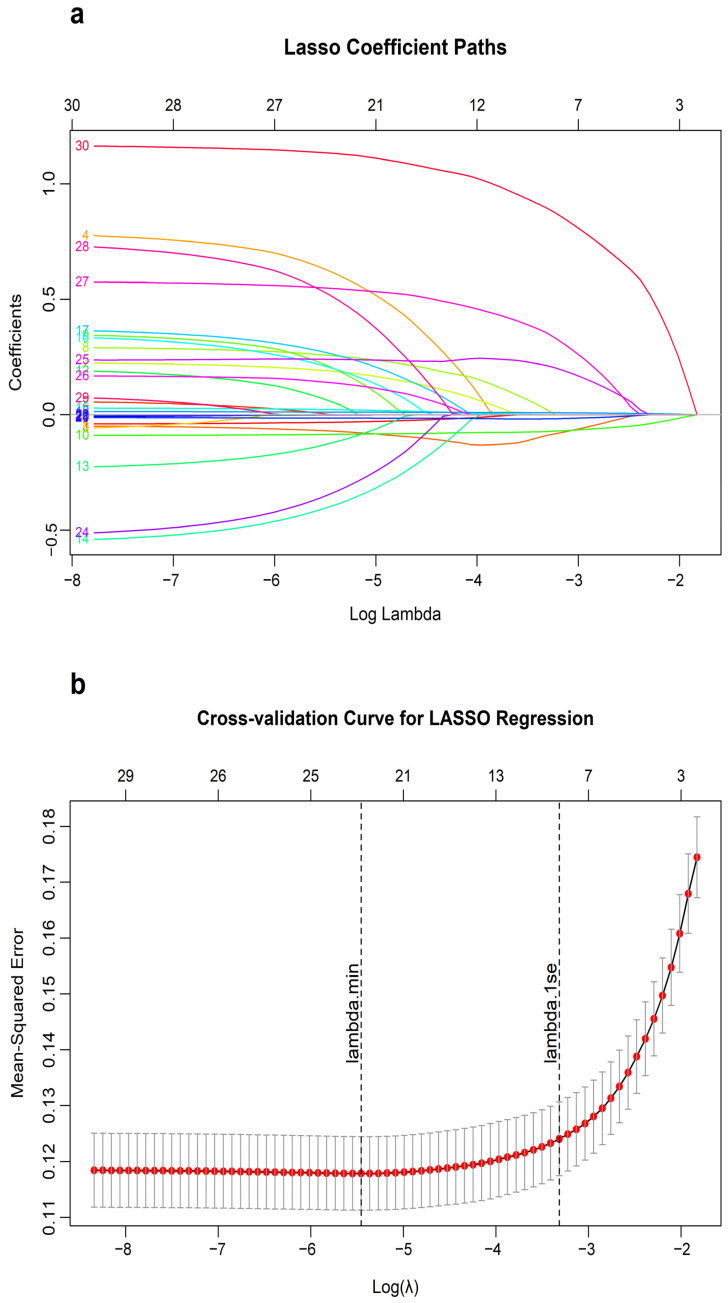
Predictor selection using the LASSO regression. Notes: (**a**) Coefficient profiles of all 30 candidate predictors plotted against the log(lambda) sequence. (**b**) 10-fold cross-validation curve showing the selection of the optimal lambda (λ) using the one-standard-error (λ.1se) criterion, resulting in 8 non-zero coefficients. Legends: The numbers 1 to 30 in panel (**a**) represent the candidate predictors. 1, Surgical age; 2, Gender; 3, Body weight; 4, Premature delivery; 5, Emergency operation; 6, Fast-track cardiac anesthesia; 7, American Society of Anesthesiologists Physical Status Classification System; 8, Risk Adjustment for Congenital Heart Surgery, version 1; 9, Previous history of cardiac surgery; 10, Left ventricular end-diastolic diameter; 11, Left ventricular fractional shortening; 12, Left ventricular ejection fraction; 13, Pulmonary arterial hypertension; 14, Red blood cell; 15, Hemoglobin; 16, White blood cell; 17, N-terminal pro B-type natriuretic peptide; 18, CPB duration; 19, Aortic clamp duration; 20, Lowest CPB temperature; 21, Lowest hematocrit during CPB; 22, Intraoperative urine output; 23, Ultrafiltrate volume; 24, Preoperative ventilatory support; 25, Continued mechanical ventilation within the first 24 postoperative hours; 26, Red blood cell transfusion volume; 27, Plasma transfusion; 28, Dopamine dose; 29, Milrinone dose; 30, Epinephrine dose.

LASSO regression reduced the initial 30 candidate variables to 8 predictors with non-zero coefficients (Figure 2). These included body weight, left ventricular end-diastolic diameter (LVDD), epinephrine dosage, CPB duration, continued mechanical ventilation within the first 24 postoperative hours, plasma transfusion, RACHS-1 category, and lowest CPB temperature.

### 3.3. Model Development and Performance Comparison

**Figure 3:**
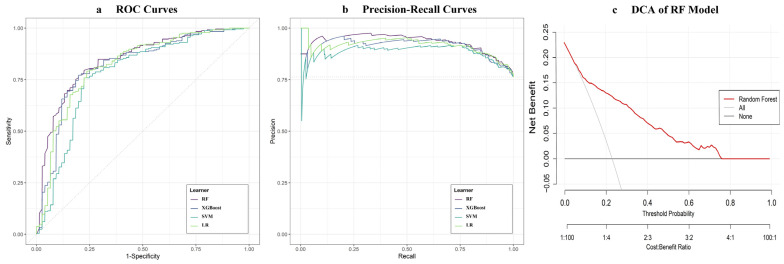
Comparative performance of ML models for POHL prediction in the validation cohort. Notes: (**a**) ROC curves and (**b**) Precision-Recall curves for LR, SVM, RF, and XGBoost. (**c**) DCA of the RF model, showing net clinical benefit across probability thresholds. The “All” and “None” lines represent strategies of treating all patients or none, respectively. Abbreviations: ROC, Receiver operating characteristic; LR, Logistic regression; SVM, Support vector machine; RF, Random forest; XGBoost, eXtreme Gradient Boosting; DCA, Decision curve analysis.

Four ML models (LR, SVM, RF, and XGBoost) were trained on the training set and subsequently evaluated on an independent validation set. In the validation set, the RF model demonstrated the highest discrimination, with an AUC of 0.821 (95% CI: 0.787–0.854), outperforming LR (0.819, 95% CI: 0.785–0.853), XGBoost (0.808, 95% CI: 0.773–0.842), and SVM (0.804, 95% CI: 0.768–0.839). The RF model also exhibited superior calibration, as indicated by the lowest Brier score (0.146). Regarding classification metrics, RF achieved the highest accuracy (0.808) and F1 score (0.625). Given the imbalanced outcome distribution, RF also excelled in AUPRC, indicating a stronger ability to identify POHL cases. DCA showed that the RF model provided a greater net clinical benefit than either the “treat all” or “treat none” strategies across a wide range of clinically relevant probability thresholds (0.10–0.75) (Figure 3, Table 2, and Supplementary Figure S5). Owing to its superior performance across these metrics, the RF model was selected for subsequent SHAP-based interpretability analysis.

### 3.4. Model Interpretation

**Figure 4:**
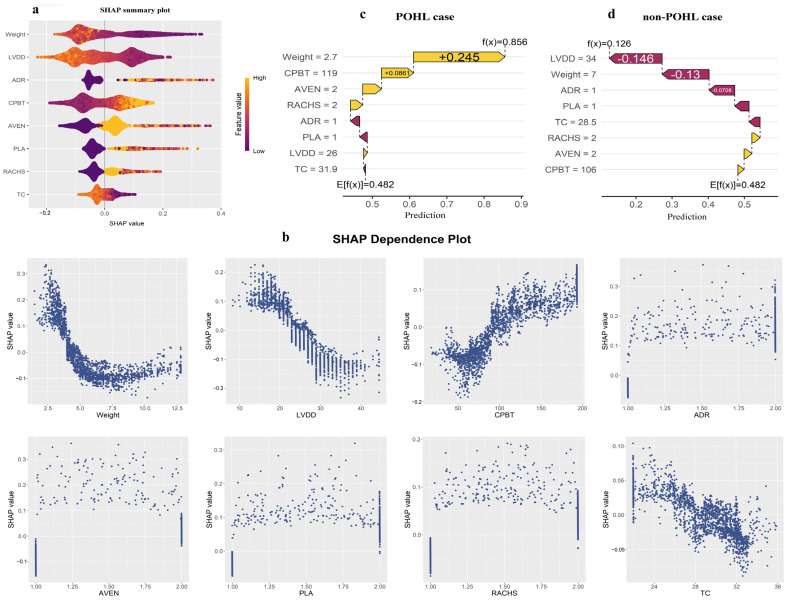
SHAP-based interpretation of the RF model for POHL prediction. Notes: (**a**) Global SHAP summary plot showing the relative importance and direction of influence of each predictor. Positive SHAP values indicate increased predicted risk; negative values indicate decreased risk. (**b**) SHAP dependence plots for selected predictors, illustrating nonlinear effects and potential clinical thresholds. (**c**,**d**) Local SHAP (waterfall) plots of two representative cases: (**c**) a high-risk POHL case where risk factors predominate, and (**d**) a low-risk non-POHL case where protective factors prevail, underscoring contrasts in individualized predictions. Yellow indicates features that increase the predicted risk (positive SHAP values), while purple indicates features with a protective effect that decrease the predicted risk (negative SHAP values). Abbreviations: SHAP, SHapley Additive exPlanation; POHL, Postoperative hyperlactatemia; CPBT, CPB duration; AVEN, Continued mechanical ventilation within the first 24 postoperative hours; ADR, Epinephrine dose; PLA, Plasma transfusion; LVDD, Left ventricular end-diastolic diameter; TC, Lowest CPB temperature; RACHS, Risk Adjustment for Congenital Heart Surgery, Version 1.

Global SHAP analysis of the final selected RF model identified several predictors associated with increased POHL risk, including higher postoperative epinephrine dose, prolonged CPB duration, continued mechanical ventilation within the first 24 postoperative hours, plasma transfusion, and higher RACHS-1 category. Conversely, greater body weight, larger LVDD, and higher intraoperative CPB temperature were consistently associated with a reduced risk (Figure 4a). These global rankings provided an overview of predictor importance and informed subsequent analyses of threshold and patient-level effects.

The SHAP dependence plots revealed nonlinear associations at clinically relevant thresholds. Children weighing less than 5 kg or undergoing CPB for more than 100 min had a substantially increased predicted risk, whereas LVDD values above 28 mm were consistently associated with reduced risk (Figure 4b).

At the individual level, SHAP explanations illustrated contrasting feature profiles between high- and low-risk predictions. In a representative high-risk case (predicted probability = 85.6%), low body weight (2.7 kg), extended CPB duration (119 min), and continued mechanical ventilation within the first 24 postoperative hours were the predominant predictors, overshadowing modest protective influences such as higher CPB temperature and low-dose epinephrine. In contrast, a low-risk case (predicted probability = 12.6%) was characterized by protective features, including larger LVDD (34 mm) and greater body weight (7.0 kg), which suppressed residual risk contributions (Figure 4c,d).

## 4. Discussion

In the cohort of children aged 0 to 36 months following congenital heart surgery at a high-volume pediatric center in China, the incidence of POHL was 22.7%, exceeding the 10–20% reported in broader pediatric populations [9,10]. This elevated incidence likely reflects the focus on a very young age group (≤36 months) with inherently limited metabolic reserve. The critical role of lactate metabolism in cardiovascular physiology and its association with clinical outcomes provides a strong rationale for investigating POHL as a key postoperative marker [34]. Among the four ML models evaluated, the RF model demonstrated superior discrimination, calibration, and net clinical benefit. The RF model not only reproduced established risk factors for POHL, such as prolonged CPB duration, higher RACHS-1 category, intraoperative hypothermia, and higher postoperative epinephrine doses [12,15,16,17], but also factors particularly relevant to early childhood, including lower body weight, smaller LVDD, plasma transfusion, and continued mechanical ventilation within the first 24 postoperative hours. These findings underscore the multifactorial nature of POHL in young children and highlight the value of advanced predictive modeling.

The risk profile identified by the model demonstrates that the pathophysiology of POHL in young children is distinct, primarily influenced by developmental immaturity in metabolic reserve and lactate clearance [9,10,11,34]. Previous studies, which often included broader pediatric age groups and relied on traditional linear analyses [9,10,11], achieved only moderate discrimination and likely did not capture the complex, nonlinear interactions among factors specific to young children. The capacity of the RF model to analyze complex interactions [20,21,22] facilitated the identification of the critical roles of developmental and management-related factors. The inconsistency of these factors in earlier literature is attributable to methodological limitations in addressing complexity within this population, rather than to a lack of clinical relevance. These findings confirm that the risk architecture for POHL in young children is uniquely shaped by developmental physiology and perioperative challenges, rather than being a simple subset of that in older patients.

The predictors identified by the model converge on two interrelated pathophysiological pathways: limited physiological reserve and increased perioperative stress. Lower body weight and reduced LVDD directly reflect diminished metabolic and cardiac buffering capacity, thereby increasing vulnerability to lactate accumulation under surgical stress [35,36,37,38,39,40,41,42]. Greater surgical complexity, as indicated by a higher RACHS-1 category, increases the risk of low cardiac output syndrome [41,43,44], which impairs tissue perfusion and promotes anaerobic glycolysis [42,44]. Prolonged CPB duration amplifies systemic inflammatory activation and cumulative oxygen debt [3,12,16,17,45,46,47], while intraoperative hypothermia reduces enzymatic activity essential for aerobic metabolism [15,48,49]. Postoperative epinephrine infusion, via β-adrenergic stimulation, accelerates aerobic glycolysis and lactate production independently of overt ischemia [50,51]. Storage-related bioactive substances in plasma transfusions, such as histamine, can impair endothelial function and exacerbate microcirculatory dysfunction, thereby contributing to systemic inflammation and metabolic stress [15,52,53]. Continued mechanical ventilation within the first 24 postoperative hours indicates increased early postoperative physiological stress and may promote lactate accumulation by impairing oxygenation, intensifying systemic inflammation, and delaying the restoration of normal metabolic function [17,52,54,55]. These mechanisms collectively indicate that POHL in young children is a maladaptive endpoint resulting from the convergence of developmental constraints, hemodynamic insults, and iatrogenic interventions. SHAP analysis further supports this framework by quantifying clinically actionable thresholds, such as body weight less than 5 kg and CPB duration greater than 100 min, thereby bridging model predictions with bedside clinical decision-making [20,22].

These findings offer actionable insights for perioperative care. The interpretable ML framework, developed using routine clinical data, facilitates proactive risk stratification for POHL and shifts management from reactive correction to early prevention. The model identifies specific, modifiable clinical levers, such as reducing CPB duration, implementing restrictive plasma transfusion triggers, and promoting early extubation, which can be prioritized in individualized surgical plans. SHAP explanations enhance model transparency by enabling care teams to visualize the most influential risk factors for each patient and to tailor interventions accordingly. Although external validation remains necessary, this study introduces a validated and interpretable tool that advances the integration of data-driven decision support into the congenital heart surgery workflow.

## 5. Limitations and Future Directions

This single-center, retrospective study reflects the practices and patient demographics of GWCMC in southern China. Consequently, the generalizability of these findings to regions with distinct healthcare systems or varying distributions of CHD subtypes may be limited. Multicenter validation across diverse pediatric populations is necessary to confirm the broader applicability of the model. Reliance on routinely collected data introduces potential risks of incomplete documentation and residual confounding, particularly for variables such as intraoperative perfusion quality and postoperative metabolic interventions, which were inconsistently recorded. Employing more standardized datasets and prospective data collection is required to address these limitations. While the RF model demonstrated strong internal performance, its external robustness has not yet been established in other models or clinical settings. Further research should examine the consistency of feature attribution across various ML algorithms.

Subsequent research should expand the model by incorporating longitudinal perioperative trajectories and genetic data to improve risk stratification and predictive accuracy in pediatric CHD patients. Including CHD subtype classifications and accounting for regional or racial factors may further enhance the model’s generalizability across diverse populations. Integrating the model into clinical decision support systems could facilitate earlier identification of high-risk patients and enable more individualized care. Nevertheless, multicenter validation in varied clinical settings remains essential to confirm broader applicability and ensure safe implementation in routine practice.

## 6. Conclusions

In this study, we developed and internally validated an interpretable RF model for predicting POHL in children aged 0 to 36 months after congenital heart surgery. The model achieved strong discrimination and calibration, and SHAP analysis enhanced interpretability by elucidating both established and novel predictors. These findings demonstrate the utility of interpretable ML in capturing complex perioperative risk profiles.

## Figures and Tables

**Table 1:** Comparison of demographic and clinical characteristics between POHL and Non-POHL pediatric patients, and between training and validation sets.

Characteristics	Total (*n* = 3224)	Non-POHL (*n* = 2493)	POHL (*n* = 731)	*p*-Value	Training Set (*n* = 2260)	Validation Set (*n* = 964)	*p*-Value
Group [n (%)]							0.8
Non-POHL	2493 (77.3)				1750 (77.4)	743 (77.1)	
POHL	731 (22.7)				510 (22.6)	221 (22.9)	
Age (months) [median (IQR)]	5 (2, 11)	6 (3, 12)	1 (0, 5)	<0.001	5 (2, 11)	5 (2, 10)	>0.9
Gender [n (%)]				0.010			0.9
Female	1405 (43.6)	1117 (44.8)	288 (39.4)		987 (44%)	418 (43%)	
Male	1819 (56.4)	1376 (55.2)	443 (60.6)		1273 (56%)	546 (57%)	
Weight (kg) [median (IQR)]	5.70 (4.30, 7.70)	6.1 (4.8, 8.0)	4.0 (3.2, 5.8)	<0.001	5.70 (4.30, 7.70)	5.70 (4.20, 7.60)	0.4
PTL [n (%)]				<0.001			0.3
No	3172 (98.4)	2474 (99.2)	698 (95.5)		2227 (98.5)	945 (98.0)	
Yes	52 (1.6)	19 (0.8)	33 (4.5)		33 (1.5)	19 (2.0)	
ER [n (%)]				0.003			0.8
No	2867 (88.9)	2239 (89.8)	628 (85.9)		2012 (89.0)	855 (88.7)	
Yes	357 (11.1)	254 (10.2)	103 (14.1)		248 (11.0)	109 (11.3)	
FTCA [n (%)]				<0.001			0.6
No	2026 (62.8)	1393 (55.9)	633 (86.6)		1414 (62.6)	612 (63.5)	
Yes	1198 (37.2)	1100 (44.1)	98 (13.4)		846 (37.4)	352 (36.5)	
ASA [n (%)]				<0.001			0.074
I~II	1297 (40.2)	1158 (46.5)	139 (19.0)		932 (41.2)	365 (37.9)	
III~VI	1927 (59.8)	1335 (53.5)	592 (81.0)		1328 (58.8)	599 (62.1)	
RACHS-1 [n (%)]				<0.001			0.7
I~II	2155 (66.8)	1813 (72.7)	342 (46.8)		1515 (67.0)	640 (66.4)	
III~VI	1069 (33.2)	680 (27.3)	389 (53.2)		745 (33.0)	324 (33.6)	
CSH [n (%)]				0.033			0.8
No	3107 (96.4)	2412 (96.8)	695 (95.1)		2177 (96.3)	930 (96.5)	
Yes	117 (3.6)	81 (3.2)	36 (4.9)		83 (3.7)	34 (3.5)	
LVDD (mm) [median (IQR)]	27 (22, 31)	28 (24, 32)	21 (18, 26)	<0.001	27 (22, 31)	27 (22, 31)	0.2
FS (%) [median (IQR)]	35.0 (32.0, 38.0)	35.0 (32.0, 38.0)	34.0 (31.0, 38.0)	0.003	35.0 (32.0, 38.0)	35.0 (32.0, 38.0)	0.9
LVEF [n (%)]				0.005			0.069
EF ≥ 50%	3179 (98.6)	2466 (98.9)	713 (97.5)		2234 (98.8)	945 (98.0)	
EF < 50%	45 (1.4)	27 (1.1)	18 (2.5)		26 (1.2)	19 (2.0)	
PAH [n (%)]				0.075			0.3
No	1782 (55.3)	1399 (56.1)	383 (52.8)		1264 (55.9)	518 (53.7)	
Yes	1442 (44.7)	1094 (43.9)	348 (47.6)		996 (44.1)	446 (46.3)	
RBC (×10^12^/L) [median (IQR)]	4.28 (3.72, 4.77)	4.33 (3.82, 4.75)	4.05 (3.43, 4.91)	<0.001	4.28 (3.72, 4.77)	4.28 (3.72, 4.80)	0.6
HGB (g/L) [median (IQR)]	113 (102, 125)	111 (101, 123)	118 (105, 138)	<0.001	113 (102, 125)	112 (102, 125)	0.8
WBC [n (%)]				0.006			0.2
WBC < 15 × 10^12^/L	2932 (90.9)	2286 (91.7)	646 (88.4)		2064 (91.3)	868 (90.0)	
WBC ≥ 15 × 10^12^/L	292 (9.1)	207 (8.3)	85 (11.6)		196 (8.7)	96 (10.0)	
NT-proBNP [n (%)]				<0.001			>0.9
NT-proBNP ≤ 250 pg/mL	682 (21.2)	609 (24.4)	73 (10.0)		479 (21.2)	203 (21.1)	
NT-proBNP > 250 pg/mL	2542 (78.8)	1884 (75.6)	658 (90.0)		1781 (78.8)	761 (78.9)	
CPBT (min) [median (IQR)]	82 (64, 116)	76 (61, 102)	116 (83, 156)	<0.001	83 (64, 116)	80 (64, 116)	0.7
ACCT (min) [median (IQR)]	46 (34, 64)	44 (32, 58)	60 (42, 85)	<0.001	46 (34, 64)	46 (34, 65)	0.8
TC (°C) [median (IQR)]	30.6 (28.0, 32.0)	31.0 (29.0, 32.0)	28.0 (25.5, 30.7)	<0.001	30.7 (28.0, 32.0)	30.5 (28.0, 32.0)	0.5
Hct (%) [median (IQR)]	23.0 (21.0, 25.0)	23 (21, 25)	23 (20, 24)	0.003	23.0 (21.0, 25.0)	23.0 (21.0, 25.0)	0.4
UV (mL) [median (IQR)]	70 (30, 150)	80 (30, 150)	50 (20, 140)	<0.001	70 (30, 150)	70 (30, 150)	0.5
UFV (mL) [median (IQR)]	450 (300, 600)	450 (350, 600)	400 (300, 550)	0.002	450 (350, 600)	450 (300, 600)	0.5
BVEN [n (%)]				<0.001			0.059
No	2927 (90.8)	2311 (92.7)	616 (84.3)		2066 (91.4)	861 (89.3)	
Yes	297 (9.2)	182 (7.3)	115 (15.7)		194 (8.6)	103 (10.7)	
AVEN [n (%)]				<0.001			0.061
No	1680 (52.1)	1509 (60.5)	171 (23.4)		1202 (53.2)	478 (49.6)	
Yes	1544 (47.9)	984 (39.5)	560 (76.6)		1058 (46.8)	486 (50.4)	
RBCT [n (%)]				<0.001			0.3
RBCT ≤ 1 u	2632 (81.6)	2117 (84.9)	515 (70.5)		1855 (82.1)	777 (80.6)	
RBCT > 1 u	592 (18.4)	376 (15.1)	216 (29.5)		405 (17.9)	187 (19.4)	
PLA [n (%)]				<0.001			0.8
No	2764 (85.7)	2280 (91.5)	484 (66.2)		1940 (85.8)	824 (85.5)	
Yes	460 (14.3)	213 (8.5)	247 (33.8)		320 (14.2)	140 (14.5)	
DOP [n (%)]				<0.001			>0.9
DOP ≤ 10 μg/kg/min	3191 (99.0)	2479 (99.4)	712 (97.4)		2237 (99.0)	954 (99.0)	
DOP > 10 μg/kg/min	33 (1.0)	14 (0.6)	19 (2.6)		23 (1.0)	10 (1.0)	
MILI [n (%)]				<0.001			0.4
MILI ≤ 0.75 μg/kg/min	2998 (93.0)	2339 (93.8)	659 (90.2)		2107 (93.2)	891 (92.4)	
MILI > 0.75 μg/kg/min	226 (7.0)	154 (6.2)	72 (9.8)		153 (6.8)	73 (7.6)	
ADR [n (%)]				<0.001			0.5
ADR ≤ 0.1 μg/kg/min	2921 (90.6)	2396 (96.1)	525 (71.8)		2043 (90.4)	878 (91.1)	
ADR > 0.1 μg/kg/min	303 (9.4)	97 (3.9)	206 (28.2)		217 (9.6)	86 (8.9)	

Footnotes: Continuous variables are presented as median with IQR, and categorical variables are presented as counting (*n*) and percentage (%). Abbreviations: PTL, Premature delivery; ER, Emergency operation; FTCA, Fast-track cardiac anesthesia; ASA, American Society of Anesthesiologists Physical Status Classification System; RACHS-1, Risk Adjustment for Congenital Heart Surgery, version 1; CSH, Previous history of cardiac surgery; LVDD, Left ventricular end-diastolic diameter; FS, Left ventricular fractional shortening; LVEF, Left ventricular ejection fraction; PAH, Pulmonary arterial hypertension; RBC, Red blood cell; HGB, Hemoglobin; WBC, White blood cell; NT-proBNP, N-terminal pro B-type natriuretic peptide; CPBT, CPB duration; ACCT, Aortic clamp duration; TC, Lowest CPB temperature; Hct, Lowest hematocrit during CPB; UV, Intraoperative urine output; UFV, Ultrafiltrate volume; BVEN, Preoperative ventilatory support; AVEN, Continued mechanical ventilation within the first 24 postoperative hours; RBCT, Red blood cell transfusion volume; PLA, Plasma transfusion; DOP, Dopamine dose; MILI, Milrinone dose; ADR, Epinephrine dose.

**Table 2:** Predictive performance of the four ML models.

Metric	LR	SVM	RF	XGBoost
AUC (95% CI)	0.819 (0.785, 0.853)	0.804 (0.768, 0.839)	0.821 (0.787, 0.854)	0.808 (0.773, 0.842)
Accuracy	0.776	0.786	0.808	0.791
Sensitivity	0.751	0.697	0.697	0.656
Specificity	0.783	0.813	0.841	0.832
PPV	0.508	0.526	0.566	0.537
NPV	0.914	0.900	0.903	0.890
F1 score	0.606	0.599	0.625	0.591
Brier score	0.166	0.161	0.146	0.153

Abbreviations: AUC, Area under the curve; PPV, Positive predictive value; NPV, Negative predictive value; LR, Logistic regression; RF, Random forest; SVM, Support vector machine; XGBoost, eXtreme Gradient Boosting.

## Data Availability

Due to privacy regulations and ethical standards, the raw data are not publicly available. De-identified datasets may be provided upon reasonable request to the authors, subject to approval by the first author, Yuchan Chen, and the corresponding author, Yajun Chen.

## Supplementary Materials

The following supporting information can be downloaded at: https://www.mdpi.com/article/10.3390/jcm15051846/s1, Figure S1: Identification and winsorization of outliers in continuous variables; Figure S2: Proportion of missing data across variables; Figure S3: Class balancing procedure for the training dataset using a hybrid resampling strategy; Figure S4: Two-dimensional t-SNE plot of the training set after hybrid resampling; Figure S5: Row-normalized confusion matrices for the four ML models in the validation cohort; Table S1: Definitions, units, and coding of variables used in model development; Table S2: The optimal hyperparameters of ML models determined by Grid Search with 10-fold CV.

## Abbreviations

ASA	American society of anesthesiologists
AUC	Area under the curve
CHD	Congenital heart disease
CPB	Cardiopulmonary bypass
DCA	Decision curve analysis
IQR	Interquartile range
LASSO	Least Absolute Shrinkage and Selection Operator
LR	Logistic regression
LVDD	Left ventricular end-diastolic diameter
ML	Machine learning
POHL	Postoperative hyperlactatemia
RACHS-1	Risk Adjustment for Congenital Heart Surgery, Version 1
RF	Random forest
ROC	Receiver operating characteristic
SHAP	SHapley Additive exPlanation
SMOTE	Synthetic minority oversampling technique
SVM	Support vector machine
XGBoost	eXtreme Gradient Boosting
