# Supplementary material for: Development and Validation of an Interpretable Model for Predicting Postoperative Hyperlactatemia in Young Children Following Congenital Heart Surgery
Source: J Clin Med. 2026 Feb 28;15(5):1846. doi: 10.3390/jcm15051846 (PMC12986052; doi:10.3390/jcm15051846)
Supplement: Supplementary file 1 [file jcm-15-01846-s001.zip › jcm-4114906-supplementary.pdf]

Supplementary Materials

Development and Validation of an Interpretable Model for Predicting Postoperative Hyperlactatemia in Young Children Following Congenital Heart Surgery

Yuchan Chen [1,2,†]; Wenxin Ge [1,†]; Lixin Hu [1]; Jiaqi Chen [1]; Yajun Chen [1,*].

[1] Department of Maternal and Child Health, School of Public Health, Sun Yat-sen University, No.74 Zhongshan 2nd Road, Yuexiu District, Guangzhou 510080, China

[2] Cardiac Intensive Care Unit, Guangzhou Women and Children's Medical Center, Guangzhou Medical University, Guangdong Provincial Clinical Research Center for Child Health, Guangzhou 510623, China

*** Correspondence: chenyj68@mail.sysu.edu.cn**

† These authors contributed equally to this work.

Table of Contents

Supplementary Figure S1 Identification and winsorization of outliers in continuous variables 3

Supplementary Figure S2 Proportion of missing data across variables........ 4

Supplementary Figure S3 Class balancing procedure for the training dataset using a hybrid resampling strategy........ 5

Supplementary Figure S4 Two-dimensional t-SNE plot of the training set after hybrid resampling 6

Supplementary Figure S5 Row-normalized confusion matrices for the four ML models in the validation cohort........ 7

Supplementary Table S1 Definitions, units, and coding of variables used in model development 8

Supplementary Table S2 The optimal hyperparameters of ML models determined by Grid Search with 10-fold CV........ 9

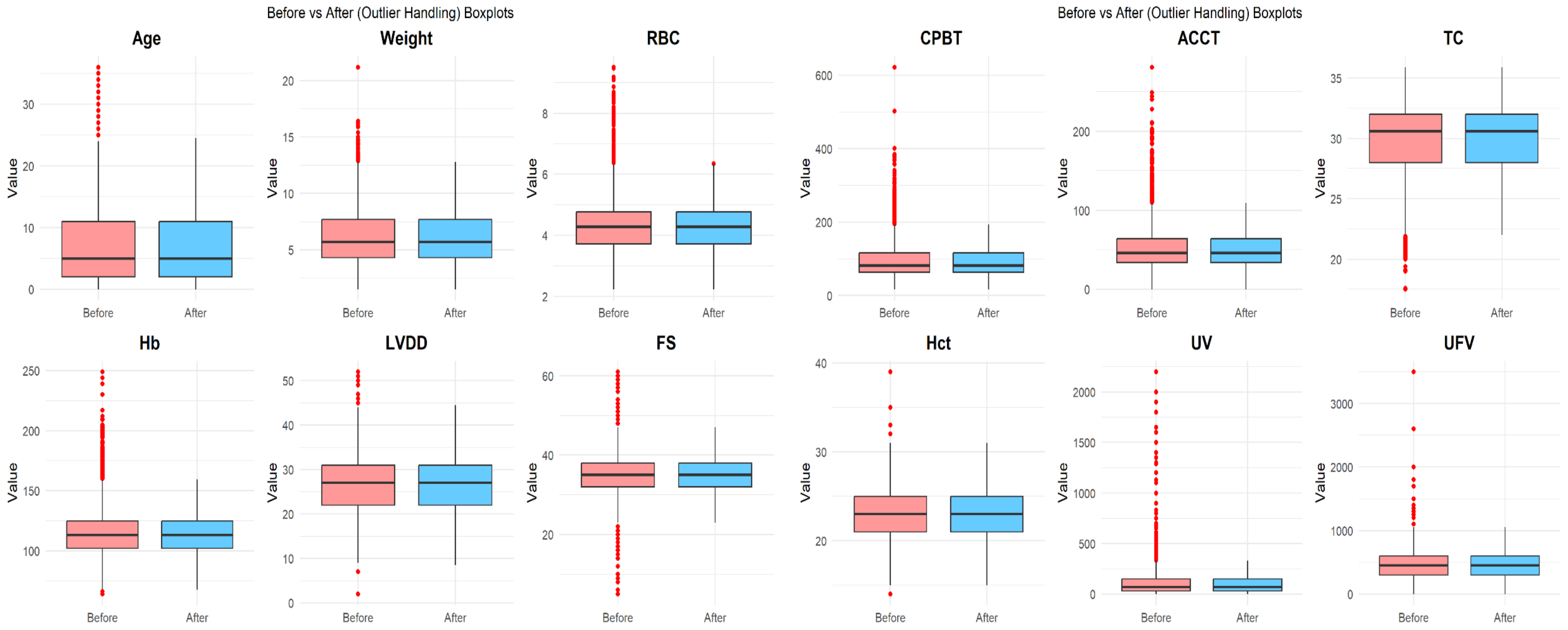

Supplementary Figure S1 Identification and winsorization of outliers in continuous variables

Footnotes: Outliers (indicated as red dots) were identified using the interquartile range (IQR) rule. This rule defines outliers as values that are either greater than Q3 + 1.5 × IQR or less than Q1 - 1.5 × IQR. The "Before" section illustrates the original distribution of the variables before winsorization, while the "After" section shows the distribution following winsorization, in which the nearest IQR boundary replaces extreme values.

Abbreviations: RBC, red blood cell count; Hb, Hemoglobin; LVDD, Left ventricular end-diastolic diameter; FS, Left ventricular fractional shortening; CPBT, CPB duration; ACCT, Aortic clamp duration; TC, Lowest CPB temperature; Hct, Lowest hematocrit during CPB; UV, Intraoperative urine output; UFV, Ultrafiltrate volume.

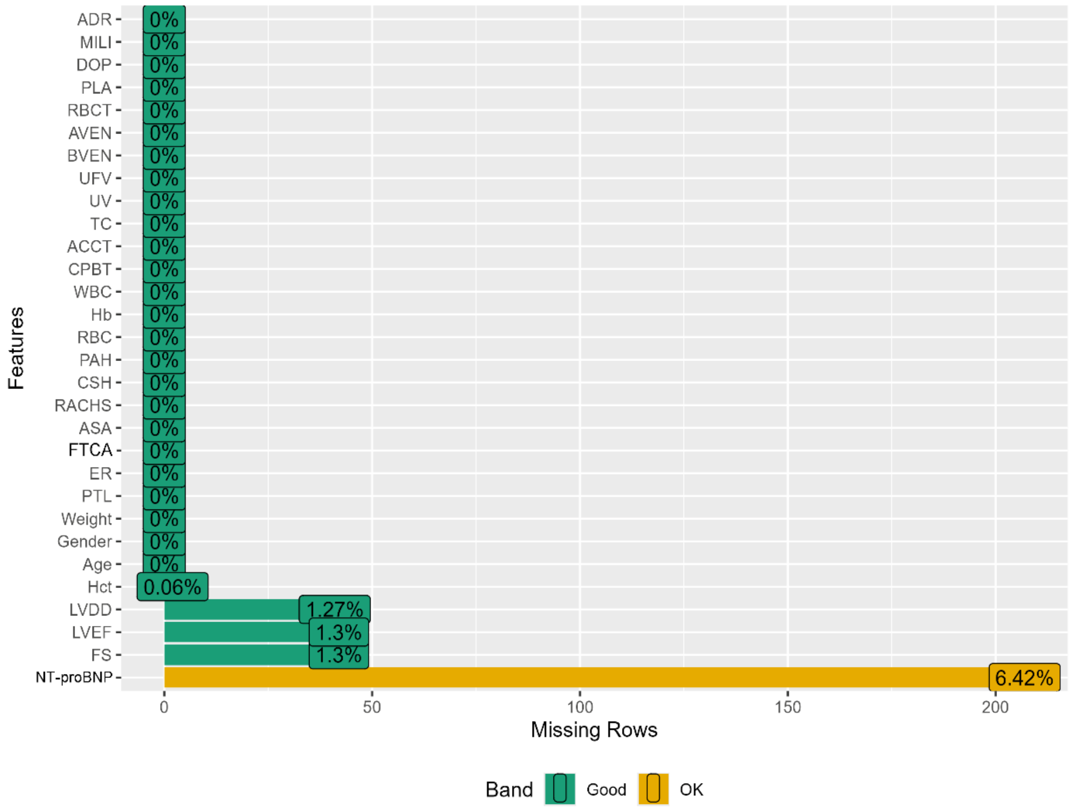

Supplementary Figure S2 Proportion of missing data across variables

Footnotes: The color bands indicate levels of data completeness: green for "good" (missingness < 5%) and yellow for "OK" (missingness ≤ 20%).

Abbreviations: PTL, Premature delivery; ER, Emergency operation; FTCA, Fast-track cardiac anesthesia; ASA, American Society of Anesthesiologists Physical Status Classification System; RACHS, Risk Adjustment for Congenital Heart Surgery, version 1; CSH, Previous history of cardiac surgery; LVDD, Left ventricular end-diastolic diameter; FS, Left ventricular fractional shortening; LVEF, Left ventricular ejection fraction; PAH, Pulmonary arterial hypertension; RBC, Red blood cell; HGB, Hemoglobin; WBC, White blood cell; NT-proBNP, N-terminal pro B-type natriuretic peptide; CPBT, CPB duration; ACCT, Aortic clamp duration; TC, Lowest CPB temperature; Hct, Lowest hematocrit during CPB; UV, Intraoperative urine output; UFV, Ultrafiltrate volume; BVEN, Preoperative ventilatory support; AVEN, Continued mechanical ventilation within the first 24 postoperative hours; RBCT, Red blood cell transfusion volume; PLA, Plasma transfusion; DOP, Dopamine dose; MILI, Milrinone dose; ADR, Epinephrine dose.

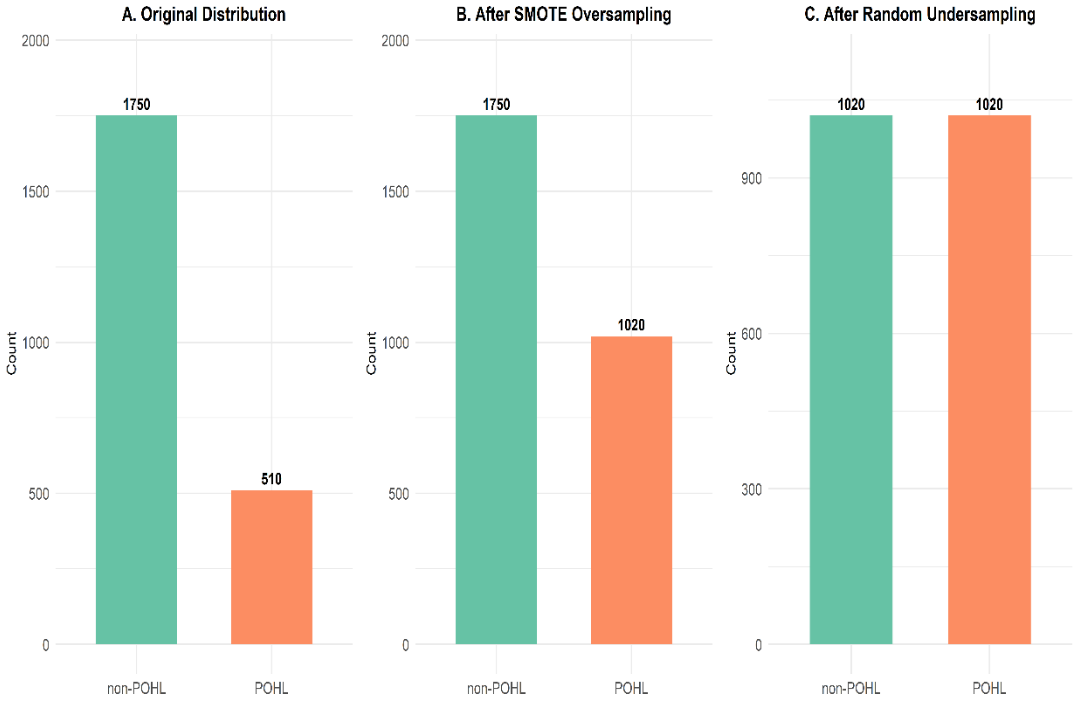

Supplementary Figure S3 Class balancing procedure for the training dataset using a hybrid resampling strategy

(A) Original training set with class imbalance; (B) SMOTE oversampling of the minority class (POHL; K = 5); (C) Random undersampling of the majority class (non-POHL), resulting in balanced classes. The validation set was retained without modification for independent evaluation.

Abbreviations: SMOTH, Synthetic Minority Over-sampling Technique; POHL, Postoperative hyperlactatemia; non-POHL, non-hyperlactatemia.

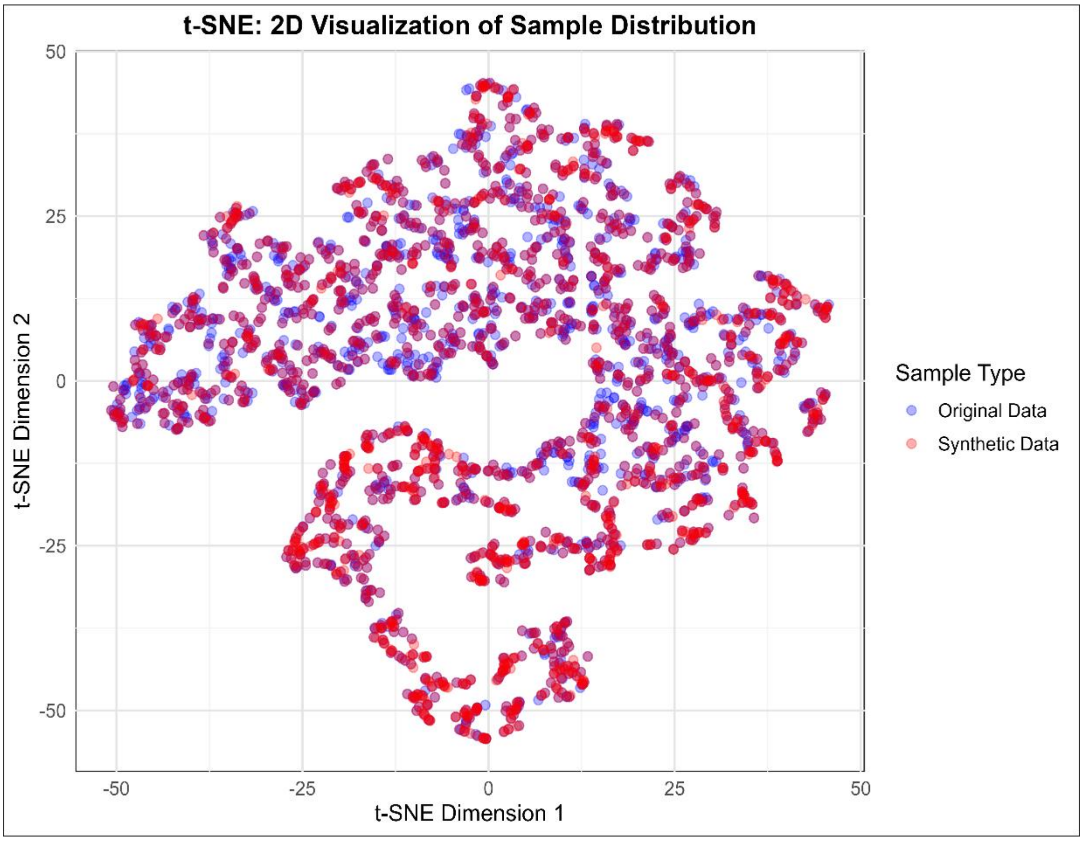

Supplementary Figure S4 Two-dimensional t-SNE plot of the training set after hybrid resampling

t-SNE was used to project the high-dimensional feature space into two dimensions. Blue points represent original samples, while red points represent synthetic samples. The close overlap of both colors suggests that the synthetic data preserves the structure of the original set.

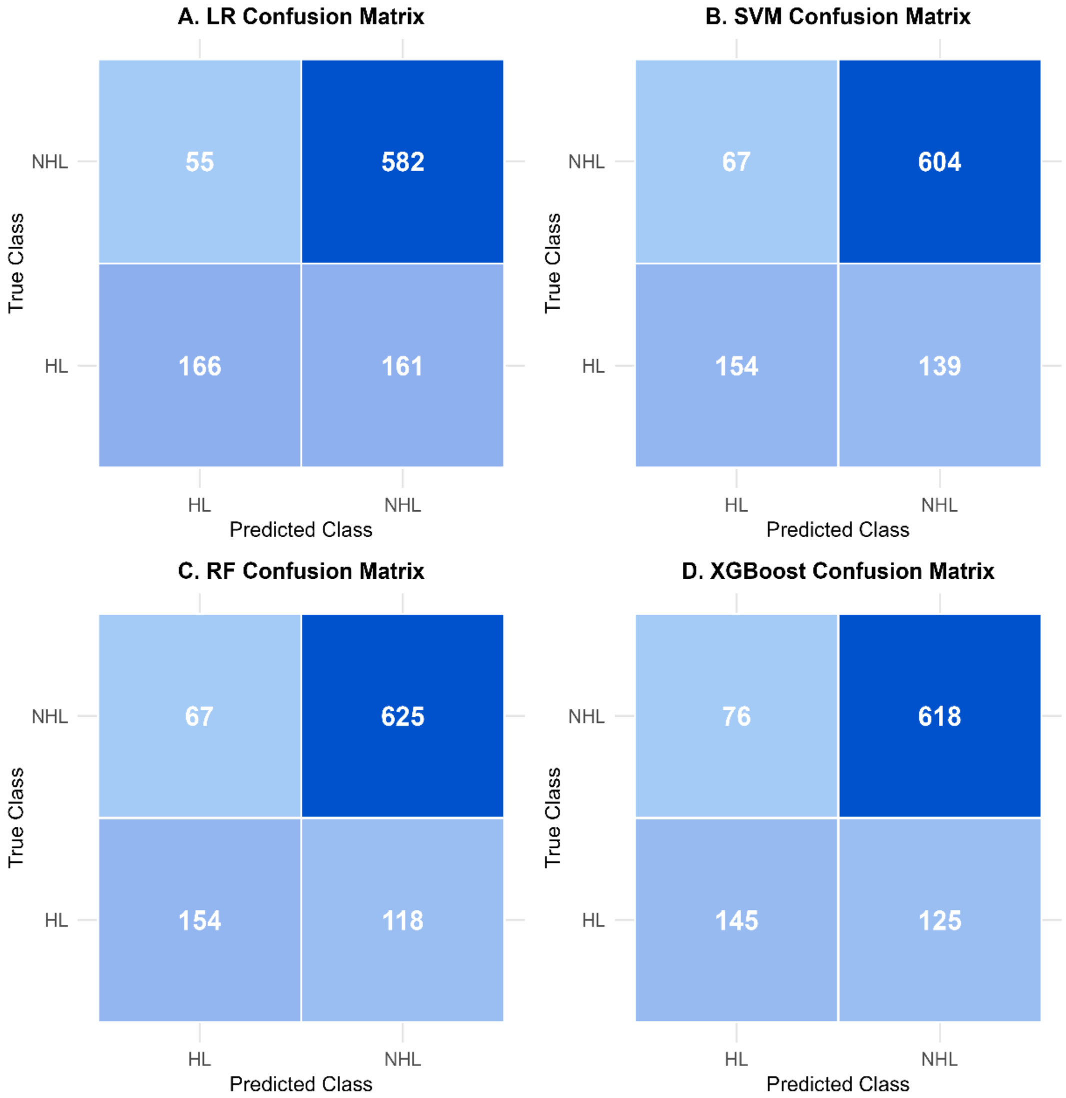

Supplementary Figure S5 Row-normalized confusion matrices for the four ML models in the validation cohort

(A) Logistic Regression (LR); (B) Support Vector Machine (SVM); (C) Random Forest (RF); (D) XGBoost. Rows correspond to observed classes and columns to predicted class (positive = postoperative hyperlactatemia, HL; negative = non-hyperlactatemia, NHL). Color intensity reflects the proportion of predictions within each observed class. Confusion matrices were generated using model-specific optimal probability thresholds determined by Youden's J statistic, which balances sensitivity and specificity, thereby allowing for a fair comparison across models.

Supplementary Table S1 Definitions, units, and coding of variables used in model development

Category	Variable	Full name	Unit/Assignment
	Group	---	0: Non-POHL<br>1: POHL
**Demographics**	Age	Surgical age	months
	Gender	Gender	0: Female<br>1: Male
	Weight	Body weight	kg
	PTL	Premature delivery	0: No<br>1: Yes
**Echocardiography findings**	LVDD	Left ventricular end-diastolic diameter	mm
	FS	Left ventricular fractional shortening	%
	EF	Left ventricular ejection fraction	0: EF ≥ 50%<br>1: EF＜50%
	PAH	Pulmonary arterial hypertension	0: No<br>1: Yes
**Laboratory parameters**	RBC	Red blood cell count	$*10^{12}/L$
	HGB	Hemoglobin	g/L
	WBC	White blood cell count	0: WBC＜$15*10^{12}/L$<br>1: WBC ≥ $15*10^{12}/L$
	NT-proBNP	N-terminal pro B-type natriuretic peptide	0: NT-proBNP≤250pg/ml<br>1: NT-proBNP＞250pg/ml
**Surgical classification**	ER	Emergency operation	0: No<br>1: Yes
	FTCA	Fast-track cardiac anesthesia	0: No<br>1: Yes
	ASA	American Society of Anesthesiologists Physical Status Classification System	0: I～II<br>1: III～VI
	RACHS-1	Risk Adjustment for Congenital Heart Surgery, version 1	0: I～II<br>1: III～VI
	CSH	Previous history of cardiac surgery	0: No<br>1: Yes
**CPB records**	CPBT	Cardiopulmonary bypass duration	min
	ACCT	Aortic clamp duration	min
	TC	Lowest cardiopulmonary bypass temperature	℃
	Hct	Lowest hematocrit during cardiopulmonary bypass	%
	UV	Intraoperative urine output	ml
	UFV	Ultrafiltrate volume	ml
**Postoperative treatment**	BVEN	Preoperative ventilatory support	0: No<br>1: Yes
	AVEN	Continued mechanical ventilation within the first 24 postoperative hours	0: No<br>1: Yes
	RBCT	Red blood cell transfusion volume (during surgery and within first 24 hours postoperative)	0: RBCT≤1u<br>1: RBCT＞1u
	PLA	Plasma transfusion (during surgery and within first 24 hours postoperative)	0: No<br>1: Yes
	DOP	Dopamine dose (maximum infusion dose within 24 hours postoperatively, excluding rescue doses)	0: DOP ≤ 10μg/kg/min<br>1: DOP＞10μg/kg/min
	MILI	Milrinone dose (maximum infusion dose within 24 hours postoperatively, excluding rescue doses)	0: MILI ≤ 0.75μg/kg/min<br>1: MILI＞0.75μg/kg/min
	ADR	Epinephrine dose (maximum infusion dose within 24 hours postoperatively, excluding rescue doses)	0: ADR ≤ 0.1μg/kg/min<br>1: ADR＞0.1μg/kg/min

Supplementary Table S2 The optimal hyperparameters of ML models determined by Grid Search with 10-fold CV

Model	Parameter optimisation range	Parameters
LR	'maxit': (25,10000)	maxit=7,507
SVM	'cost':(0.001,10), 'gamma':(0.001,1), 'kernel':(linear,radial,polynomial,sigmoid), 'degree':(1,5), 'coef':(-1,1)	cost=2.50075, gamma=0.25075, kernel=radial, degree=3, coef0=-0.5
RF	'ntree':(500), 'mtry':(1,3), 'nodesize':(1,10), 'maxnodes':(10,100)	Ntree=500, mtry=2, nodesize=10, maxnodes=100
XGBoost	'max_depth':(3,10), 'eta':(0.01,0.2),' 'nrounds':(50,300), 'min_child_weight':(1,10), 'gamma':(0,0.25), 'subsample':(0.5,1), 'colsample_bytree':(0.5,1)	max_depth=9, eta=0.0575, nrounds=238, nthread=1, verbose=0, min_child_weight=1, gamma=0.25, subsample=0.625, colsample_bytree=0.875

Abbreviations: LR, Logistic regression; RF, Random forest; SVM, Support vector machine; XGBoost, eXtreme Gradient Boosting.
